# Distinct Contributions of Tryptophan Residues within the Dimerization Domain to Nanog Function

**DOI:** 10.1016/j.jmb.2016.12.001

**Published:** 2017-05-19

**Authors:** Nicholas P. Mullin, Alessia Gagliardi, Le Tran Phuc Khoa, Douglas Colby, Elisa Hall-Ponsele, Arthur J. Rowe, Ian Chambers

**Affiliations:** 1 MRC Centre for Regenerative Medicine, Institute for Stem Cell Research, School of Biological Sciences, University of Edinburgh, 5 Little France Drive, Edinburgh EH16 4UU, UK; 2 Canada's Michael Smith Genome Sciences Centre, BC Cancer Agency, 675 West 10th Avenue, Vancouver, BC, Canada V5Z 1L3; 3 Department of Anatomy and Embryology, PhD Program in Human Biology, School of Integrative and Global Majors, University of Tsukuba, 1-1-1 Tennodai, Tsukuba, Ibaraki 305-8575, Japan; 4 NCMH Business Centre, School of Biosciences, University of Nottingham, Sutton Bonington, Leicestershire LE12 5RD, UK

**Keywords:** self-renewal, dimerization, aromatic interactions, structural core, Sox2, TFs, transcription factors, WR, tryptophan repeat, ES cells, embryonic stem cells

## Abstract

The level of the transcription factor Nanog directly determines the efficiency of mouse embryonic stem cell self-renewal. Nanog protein exists as a dimer with the dimerization domain composed of a simple repeat region in which every fifth residue is a tryptophan, the tryptophan repeat (WR). Although WR is necessary to enable Nanog to confer LIF-independent self-renewal, the mechanism of dimerization and the effect of modulating dimerization strength have been unclear. Here we couple mutagenesis with functional and dimerization assays to show that the number of tryptophans within the WR is linked to the strength of homodimerization, Sox2 heterodimerization and self-renewal activity. A reduction in the number of tryptophan residues leads initially to a gradual reduction in activity before a precipitous reduction in activity occurs upon reduction in tryptophan number below eight. Further functional attrition follows subsequent tryptophan number reduction with substitution of all tryptophan residues ablating dimerization and self-renewal function completely. A strong positional influence of tryptophans exists, with residues at the WR termini contributing more to Nanog function, particularly at the N-terminal end. Limited proteolysis demonstrates that a structural core of Nanog encompassing the homeodomain and the tryptophan repeat can support LIF-independent colony formation. These results increase understanding of the molecular interactions occurring between transcription factor subunits at the core of the pluripotency gene regulatory network and will enhance our ability to control pluripotent cell self-renewal and differentiation.

## Highlights

• The Nanog tryptophan repeat (WR) is required for LIF-independent mouse embryonic stem cell self-renewal.
• Tryptophans within WR give a graded increase in self-renewal and dimerization.
• Tryptophans at the WR extremities contribute disproportionally to Nanog function.
• A structural core of Nanog containing WR can drive LIF-independent self-renewal.
• Results clarify the contribution of tryptophan residues within WR to Nanog function.

## Graphical Abstract

**Figure:**
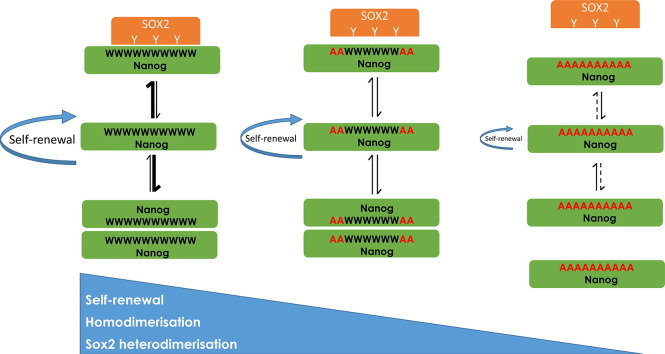

## Introduction

The processes by which cell fate decisions are made during development are controlled by a temporally and spatially organized hierarchy of transcription factors (TFs) that control gene expression and determine a cell's state. The ability of TFs to mediate these processes relies on their ability to interact with DNA in a sequence specific manner and to interact specifically with other molecules to mediate downstream effects.

Although different TF families recognize DNA and protein partners in different ways, one feature common to many TFs is their ability to form homo- or heterodimers [1]. TF dimerization has a number of functional implications. Bringing two DNA binding regions together can alter or enhance DNA binding specificity. Moreover, dimerization can create contiguous protein surfaces absent from monomers. The transition between monomers and dimers can also be regulated by post-translational modification. For example, Stat protein phosphorylation causes dimerization and nuclear translocation [2]. In contrast, estrogen receptor A phosphorylation blocks both dimerization and DNA target binding [3]. For these reasons, the identification of TF dimeric interfaces and the dissection of mechanisms by which dimerization is controlled are important to understand TF function.

In embryonic stem cells (ES cells), a central network of TFs is responsible for the maintenance of ES cell identity. This pluripotency gene regulatory network includes a number of TFs at the core of which is the triumvirate of Nanog, Oct4 and Sox2 [4], [5], [6], [7], [8], [9]. Structural information exists for the DNA binding domains for each of these three factors [10], [11], [12]. However, although it is known that each of the three proteins can form homo-multimers [13], [14], [15], [16], biophysical and structural characterization of the full-length proteins is relatively limited.

The homotypic interaction of Nanog has been characterized in most detail with mouse Nanog shown to exist in solution as a dimer [13], [16]. Homodimerization of Nanog is mediated by a region of the protein containing 10 copies of a pentapeptide repeat in which a tryptophan residue is conserved at the same position within each repeat (the tryptophan repeat, or WR) [13], [16]. Deletion of the WR from Nanog produces a molecule that cannot confer the defining biochemical property of Nanog, LIF-independent self-renewal [13]. However, the contribution of individual residues to dimerization and cellular function remains unclear. To address these issues, a series of Nanog mutants in the dimerization domain have been constructed and their functional properties investigated.

## Results

### The number of tryptophan residues is a determinant of Nanog activity

**Fig. 1:**
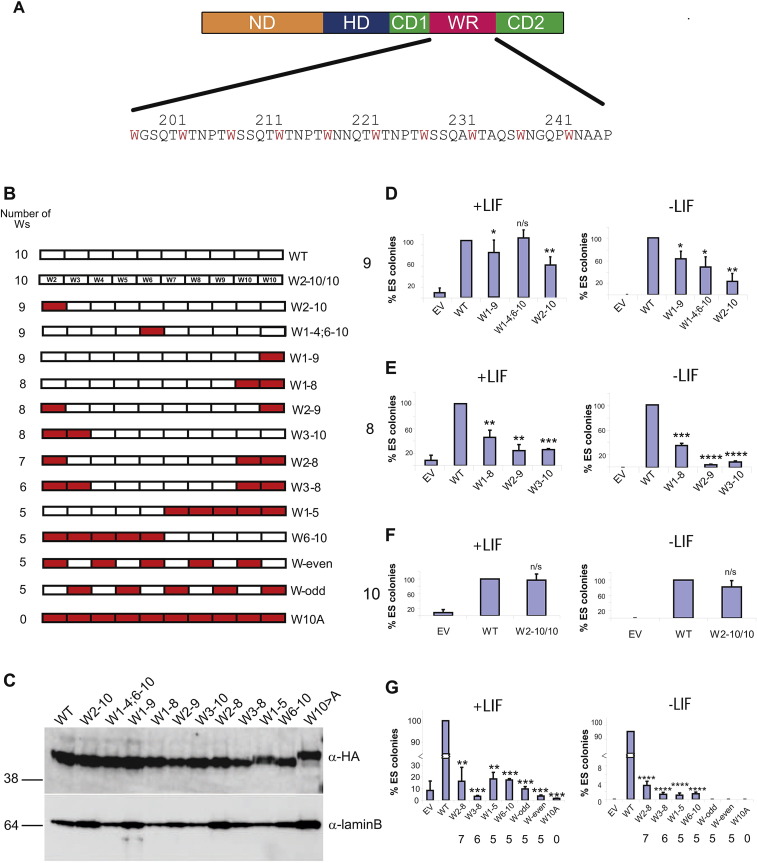
Mutation of WR tryptophan residues reduces Nanog function. (A) Cartoon representation of Nanog primary structure and sequence of WR. ND, N-terminal domain; HD, homeodomain; CD1, C-terminal domain 1; WR, tryptophan repeat; and CD2, C-terminal domain 2. W residues in the WR sequence are highlighted in red. (B) Representation of WR mutations. Each rectangle represents a 5-aa WR repeat. White blocks, wild type; filled blocks, repeats with W to A substitution. (C) Blot of nuclear lysates from E14/T cells transfected with the indicated construct. Relative molecular weights (*M*_r_) are indicated on the left-hand side of blots (kDa). (D–G) Self-renewal assays of E14/T cells transfected with Nanog variants carrying the indicated WR mutations. Colonies were stained for alkaline phosphatase and the percentage of purely alkaline phosphatase-positive colonies was determined. All assays were performed in triplicate. Data are normalized to the level of self-renewal observed in cells transfected with wild-type Nanog. For panels D–F, the number of remaining WR tryptophans is indicated at the left. For panel G, this number is given below each column. Error bars are standard deviations from three independent experiments. *P < 0.05, **P < 0.01, ***P < 0.001, ****P < 0.0001; n/s, not significant (Student's *t* test).

A series of Nanog variants were constructed in which one or more tryptophan residues within the WR were mutated to alanine (Fig. 1A and B). The ability of these variants to alter the self-renewal capacity of E14/T ES cells following transfection of constitutive episomal expression vectors was then tested. Initially, individual tryptophan-to-alanine substitutions were assessed. In each case, the ability to confer LIF-independent self-renewal was reduced but this effect was site-specific (Fig. 1D). Replacement of the N-terminal tryptophan (W2–10) reduced the number of undifferentiated ES cells colonies by ~ 80% compared to wild-type Nanog, whereas replacement of W5 or W10 had a milder effect, reducing activity by ~ 40%, relative to wild-type Nanog. In the presence of LIF, the effects of W-A substitutions were less severe and in the case of W1–4;6–10, undetectable. Furthermore, the fold-enhancement of self-renewal by LIF was greater for W2–10 than for W1–9. These data indicate that individual tryptophan residues within the WR contribute differentially to LIF-independent ES cell self-renewal, with the tryptophan residue most proximal to the homeodomain having the greatest effect.

The importance of multiple tryptophans was next examined by replacement of two tryptophan residues (Fig. 1E). Combined replacement of W1 with either W10 (W2–9) or W2 (W3–10) reduced self-renewal activity even further, to 5% in the absence of LIF. In contrast, replacement of the two C-terminal tryptophan residues (W1–8) had a more modest effect reducing LIF-independent self-renewal activity to an extent comparable to replacement of the single N-terminal tryptophan (W2–10). However, in contrast to W2–10, W1–8 was compensated less effectively by LIF addition and less effectively than either of the other mutants retaining eight tryptophan residues (Fig. 1E). These data further highlight the important contribution of tryptophan residues at the N-terminus of the WR to ES cell self-renewal activity.

To determine whether the specific sequence of the first repeat within the WR was of greater importance than the actual number of tryptophan residues present in the WR, a mutant was constructed in which the first repeat was deleted and an additional copy of repeat 10 added to the C-terminus of the WR (W2–10/10). This mutant was more similar in function to wild-type Nanog than to either W2–10 or W1–9, suggesting that the position of the first tryptophan of the WR within the overall Nanog structure is of greater importance than the specific sequence of the first repeat within the WR (Fig. 1F).

Alanine replacement of three or more tryptophan residues resulted in further reductions in activity (Fig. 1G). Interestingly, when five tryptophan residues are removed, the differential contribution of the N-terminal tryptophan residues is no longer seen. Instead, an effect of tryptophan adjacency becomes apparent. W1–5 and W6–10 have comparable activities and both exceed the activities of W-odd and W-even, which in the absence of LIF are as negligible as that of W10A. Immunoblot analyses showed that the differential activities of Nanog mutants could not be accounted for by differing protein expression levels (Fig. 1C).

### The number of tryptophan residues determines dimerization efficiency

**Fig. 2:**
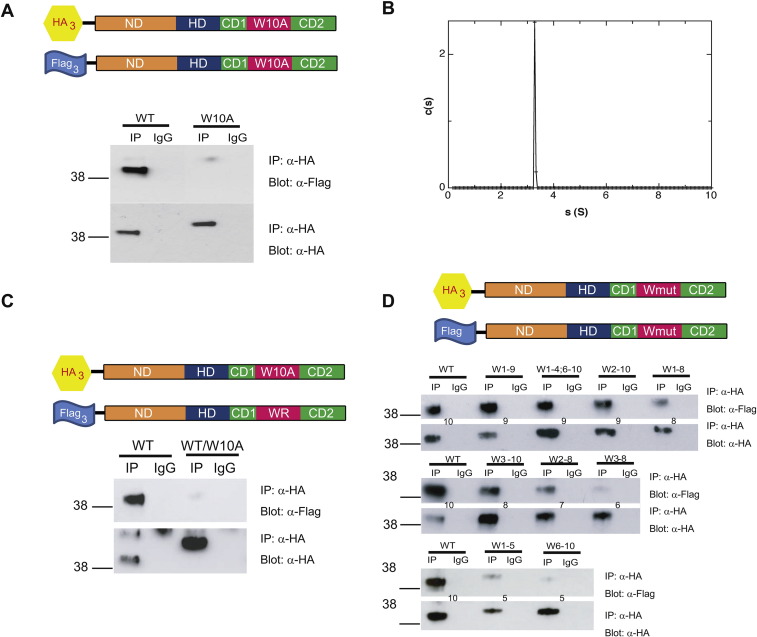
The Nanog homodimerization capacity is directly related to the number of tryptophans in the WR. (A) Co-immunoprecipitation of differentially tagged Nanog and NanogW10A. (HA)_3_Nanog and (Flag)_3_Nanog or (HA)_3_NanogW10A and (Flag)_3_NanogW10A were co-expressed in E14/T cells. The degree of interaction was assessed by immunoprecipitation with anti-HA and subsequent immunoblotting with anti-Flag. (B) Analysis of NanogW10A by AUC. Recombinant NanogW10A was analyzed by sedimentation AUC demonstrating the presence of a single species with an apparent molecular weight of 37.7 ± 2.5 kDa. (C) Co-immunoprecipitation of Nanog and NanogW10A. To assess whether the tryptophans of a single monomer are sufficient to mediate Nanog dimerization, (HA)_3_Nanog and (Flag)_3_NanogW10A were co-expressed and immunoprecipitated as described in panel A. (D) Co-immunoprecipitation of differentially tagged versions of WR mutants. Differentially tagged versions of WR mutants were co-expressed in E14/T cells and the degree of interaction assessed by immunoprecipitation as for panel A. Numbers between blots indicate the number of W residues in the mutant being tested. Numbers on the left-hand side of all blots show the positions of *M*_r._ markers (kDa).

Nanog dimerization is considered essential for LIF-independent self-renewal [13], [16]. To determine whether the loss of function observed when all tryptophan residues are substituted by alanine is reflected in a reduced dimerization ability, co-immunoprecipitation of differentially tagged versions of NanogW10A was performed. This demonstrated that substitution of all W residues in the WR abrogated dimerization capacity (Fig. 2A).

To further investigate the properties of NanogW10A, the ability of recombinant protein to dimerize was investigated using sedimentation velocity. This technique previously demonstrated that recombinant wild-type Nanog (rNanog) dimerized with a *K*_d_ in the low micromolar range [13]. Sedimentation velocity analysis showed a single peak at 3.29S (Fig. 2B). From the empirical equation of Squire and Himmel [17], this corresponds to a protein of molecular weight 37.7 ± 2.5 kDa. As this is in close agreement with the computed formula weight of a Nanog monomer (35.9 kDa), this establishes that NanogW10A is monomeric.

Although the Nanog–Nanog interaction is mediated by tryptophans within the WR, the nature of the interaction between monomers is unclear. Two possible types of contact exist in a homotypic interaction: one in which side chains of the same identity on each monomer interact and another in which side chains on one monomer interact with distinct side chains on the other. If Nanog dimerizes by homotypic tryptophan interactions, then differentially tagged Nanog and NanogW10A should not co-immunoprecipitate. In contrast, if dimerization occurs by interaction of tryptophan residues on one WR with non-tryptophan residues on the second WR, then differentially tagged Nanog and NanogW10A should co-immunoprecipitate. This was tested by co-transfection of (Flag)_3_Nanog and (HA)_3_Nanog or (HA)_3_NanogW10A (Fig. 2C). While (Flag)_3_Nanog immunoprecipitated (HA)_3_Nanog, (Flag)_3_Nanog could not immunoprecipitate (HA)_3_NanogW10A. These results indicate that Nanog dimerization involves tryptophan–tryptophan interactions most likely through stacking of the aromatic rings.

To determine whether the decrease in function observed upon tryptophan substitution is reflected in a reduced homodimerization ability, co-immunoprecipitation of differentially tagged versions of Nanog was performed. These assays demonstrate that as the number of tryptophans decreases, so does the dimerization capacity (Fig. 2D). This is clearly noticeable in mutants containing eight tryptophans (W1–8, W3–10), with dimerization further decreasing when the tryptophan number is reduced to seven (W2–8). A further reduction in tryptophan number to six (W3–8) produces a level of homodimerization that is barely detectable and comparable to mutants with only five tryptophan residues (W1–5, W6–10).

The above results suggest that the ability to dimerize is directly related to the number of tryptophans and thereby to functional activity. Indeed, it is noteworthy that mutants with the nine tryptophans do not have the same level of dimerization and activity. Comparison of W1–9 and W2–10 demonstrates that the N-terminal tryptophan contributes more to dimerization than the C-terminal tryptophan, mirroring results seen in LIF-independent self-renewal assays.

### WR tryptophans mediate Nanog–Sox2 heterodimerization

**Fig. 3:**
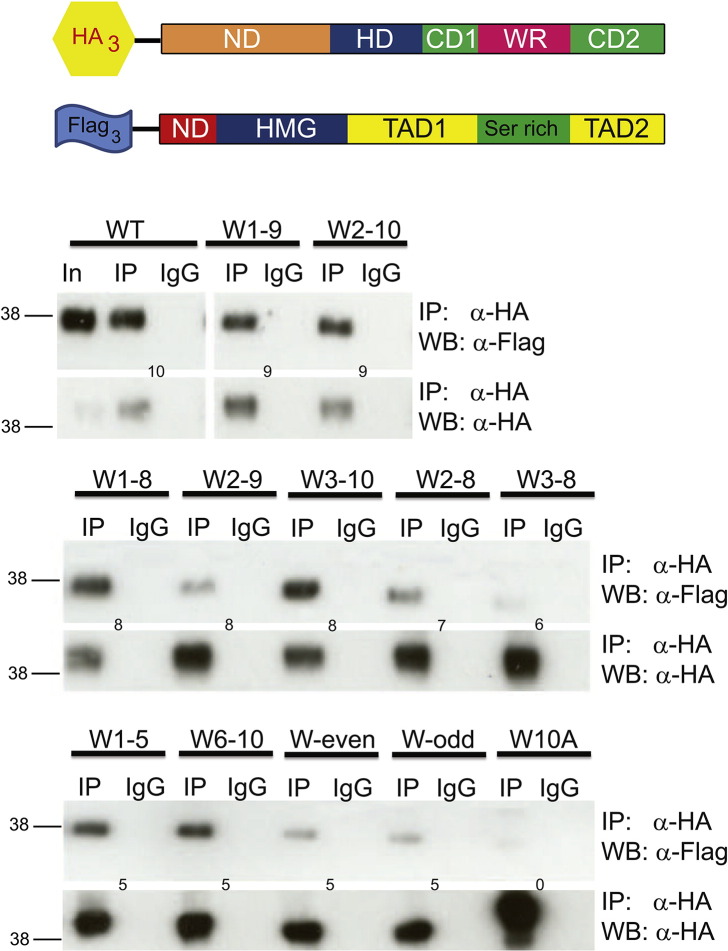
The Nanog–Sox2 interaction depends on tryptophans within the Nanog WR. Co-immunoprecipitation of Nanog variants with Sox2. (HA)_3_-Nanog variants were co-transfected with (Flag)_3_-Sox2 in E14/T cells. The degree of interaction was assessed by immunoprecipitation with anti-HA and subsequent immunoblotting with anti-Flag antibodies. Numbers between blots indicate the number of W residues in the mutant being tested. Numbers on the left-hand side of all blots show the positions of *M*_r._ markers (kDa).

We have previously demonstrated a direct, robust interaction between Nanog and Sox2 proteins which is abrogated when all tryptophans of the WR are mutated to alanine [18]. The role of tryptophan residues within the WR in heterodimerization with Sox2 (Fig. 3) was investigated using the panel of Nanog mutants (Fig. 1B). The results demonstrate that no single tryptophan residue can be assigned as the sole Sox2 binding site. The mutant Nanog harboring the central six tryptophans of the WR (W3–8) has a similar Sox2 binding capacity to W10A in which all WR tryptophans are substituted by alanine. This level of Sox2 interaction is lower than observed with any of the Nanog mutants containing only five W residues. Notably W1–5 and W6–10 both show higher Sox2 binding, suggesting that a contiguous stretch of tryptophan containing repeats at either end of WR is beneficial in engaging Sox2. This conclusion is borne out from inspection of additional results. Compared to W3–8, W2–8 shows increased Sox2 binding, suggesting that addition of the second W residue in WR to the central six W residues in W3–8 contributes to Sox2 binding. Interestingly, extension of that central group of W-containing repeats to eight by addition of W9 (in W2–9) does not markedly improve Sox2 interaction. This contrasts with the situation of the other mutants containing eight tryptophans (W1–8 and W3–10), both of which increase Sox2 binding compared to W2–8. The further stepwise increases in Sox2 binding seen when either mutant carrying nine tryptophan residues is tested or when wild-type Nanog is examined suggest that the contiguous stretches of tryptophan containing repeats at both the N- and C-termini of the WR both contribute to Sox2 binding.

### Defining a structural core of Nanog

**Fig. 4:**
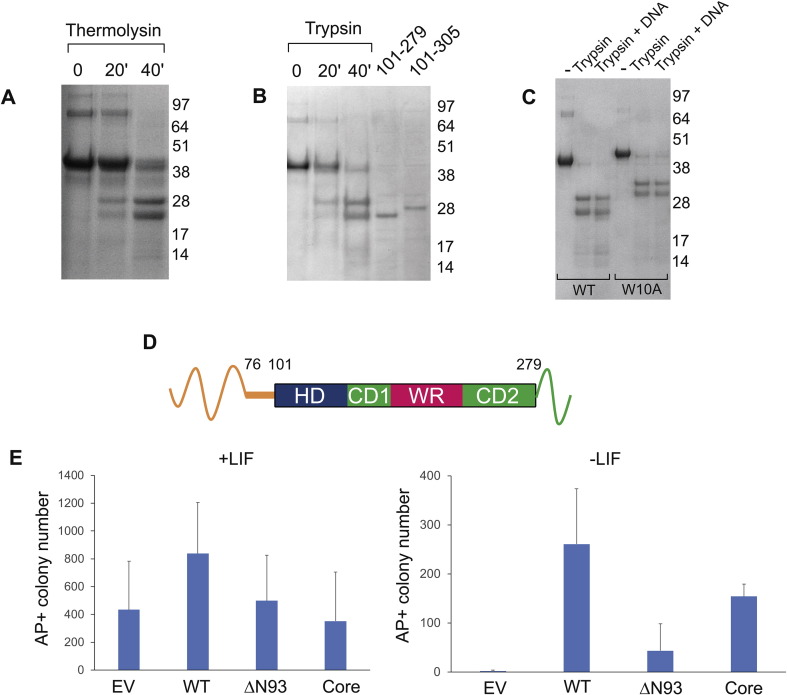
Mutation of WR does not alter the overall structure of Nanog. (A) rNanog was treated with limiting amounts of thermolysin and the resulting digestions analyzed by SDS-PAGE. Incubation times are indicated above lanes (minutes). (B) rNanog was treated with limiting amounts of trypsin and digestions analyzed by SDS-PAGE. Lanes 1–3 rNanog treated with trypsin. Incubation times are denoted above lanes (minutes). Lane 4, Nanog residues 101–279 expressed in *E.coli*. Lane 5, Nanog residues 101–305 expressed in *E.coli*. (C) Tryptic digest of rNanog and rNanogW10A in the presence and absence of DNA. (D) Cartoon of proteolytic resistance of Nanog. Blocks represent protease-resistant, more structured regions and numbers represent amino acids. (E) Self-renewal assays of E14/T cells transfected with the indicated Nanog variants in the presence or absence of LIF. Plates were stained for alkaline phosphate (AP) activity and the number of pure AP-positive colonies determined.

Structural data on Nanog are restricted to the homeodomain [10]. To obtain additional data on Nanog structure, the ability of proteases to identify a core structural domain of Nanog resistant to protease digestion was examined. Thermolysin digestion of rNanog produced core fragments of approximately 28 and 23 kDa (Fig. 4A), with similar sized fragments also produced by trypsin (Fig. 4B). In both digests, the 28-kDa fragment is more abundant initially, with the 23-kDa fragment becoming more prominent with time. The fact that Nanog has similar resistance to both enzymes suggests that the fragments obtained define a core structural domain of Nanog that is resistant to protease. To define the limits of this core, both bands were excised and analyzed by N-terminal sequencing [19]. This showed that the N-terminal residue of the 28-kDa band was Leu76 within the N-domain and that of the 23-kDa band was Thr101 at the N-terminus of the homeodomain. The size of the protease-resistant fragments suggests that they terminate at or close to the C-terminus. However, a recombinant fragment of Nanog encoding residues from 101 to the C-terminus (305) expressed in *E.coli* had a larger size than the 23-kDa band (Fig. 4B). The identification of the approximate position of the C-terminal end of the 23-kDa band was aided by the fact that there is only a single trypsin target site, Arg279 between residue 170 and the C-terminus. Therefore, a recombinant Nanog fragment corresponding to residues 101–279 was expressed in *E.coli* and compared to the size of the fragments obtained by proteolysis (Fig. 4B). The lower proteolytic band is the same size as the 101–279 product, demonstrating that the protease resistant core extends from residue 101 to residue 279 (Fig. 4B and D).

### The effect of the W10A substitution on Nanog structure

To assess the structural changes caused by tryptophan substitution and loss of dimerization ability, partial proteolysis was repeated using rNanog and monomeric rNanogW10A. The slightly slower mobility of rNanogW10A relative to rNanog is consistent with the apparent increase in size seen by SDS-PAGE for Gal4BD–tryptophan repeat fusions containing tryptophan–alanine substitutions [20]. The results demonstrate that rNanog and rNanogW10A have the same overall protease resistance properties, suggesting that the observed protease resistance pattern is inherent to the monomer and does not change upon dimerization (Fig. 4C). Moreover, the presence of an excess of dsDNA oligonucleotide known to bind Nanog [6] did not cause any change in the protease digestion pattern of either rNanog or rNanogW10A (Fig. 4C). Together these results argue that rNanog and rNanogW10A have similar structural properties and that the loss of LIF-independent self-renewal caused by substitution of tryptophans within the WR is not due to a gross perturbation of protein structure.

### The structural core of Nanog has self-renewal activity

To determine whether the structural core has biological activity, the core together with an N-terminal truncation were assayed for their ability to maintain self-renewal (Fig. 4E). These assays show that deletion up to residue 93 (ΔN93), close to the start of the canonical homeodomain, severely impairs function. It is therefore intriguing that the structural core itself, which has a 22-aa N-terminal extension relative to ΔN93, provides a higher self-renewal efficiency. These results demonstrate that a sequence between amino acids 76 and 93 contributes to Nanog function. The identity of these residues is currently unclear but the sequence includes serine residues 77 and 78, one of which can be phosphorylated in human embryonic kidney cells [21].

## Discussion

Like several other TFs, Nanog forms a homodimer. Removal of the WR region that mediates dimerization abolishes the capacity of Nanog to confer LIF-independent self-renewal [13]. However, the contribution of individual residues to dimerization remained unclear, as was the link between dimerization and activity. In the work described here, mutagenesis was performed to assess the contribution of tryptophan residues to the activity of Nanog. This demonstrated that reduction in the number of tryptophan residues had a graded response as measured by LIF-independent self-renewal and dimerization. Interestingly, however, tryptophan residues within the WR are not equivalent, with tryptophans at the WR termini having a more pronounced functional contribution. Furthermore, tryptophan residues at the N-terminal end of the WR contribute more to self-renewal than residues at the C-terminus. That this reflects the position of repeat 1 within the overall Nanog structure, rather than a dependence on the specific amino acid sequence of repeat 1, was indicated by the uncompromised activity of a mutant in which repeat 1 was deleted and repeat 10 duplicated, thereby maintaining the repeat number at 10. This may suggest a structure/function relationship between the tryptophan in repeat 1 and another part of Nanog. The most obvious link is to the DNA binding domain which is located 40 residues N-terminal to repeat 1. It is also notable that mutants bearing 9 (W9) or 8 (W8) tryptophan residues within the WR show differing degrees of compensation by LIF. Mutants W1–9 and W1–8 direct higher levels of LIF-independent self-renewal than other W9 or W8 mutants but show lower levels of enhancement by LIF. As both of these mutants retain N-terminal W residues within the WR, this suggests that the differential effect of LIF could be structurally constrained.

A Nanog partner protein could be involved in an interaction between the DNA binding homeodomain and the WR to mediate function. It is therefore intriguing that mapping of the interaction site of Sox2 onto Nanog demonstrates that although no single tryptophan residue within the Nanog WR can be assigned as solely responsible for mediating Sox2 binding, tryptophan residues at either end of WR are crucial for Sox2 binding. It is possible that the tryptophans at the ends of the WR mediate the interaction with Sox2 and that the loss of self-renewal activity observed when the tryptophans at the extremities of the repeat are substituted is a function of the loss of the critical Nanog–Sox2 protein–protein interaction [18]. However, mutagenesis of the tryptophans of the WR impairs LIF-independent self-renewal and reduces both Nanog homodimerization and Nanog–Sox2 heterodimerization. Therefore, uncoupling the contributions of homodimerization and heterodimerization to self-renewal is not straightforward. Nevertheless, some mutants (W1–5, W1–6) that do not support LIF-independent self-renewal show a greater reduction in homodimerization than Sox2 heterodimerization. Although this may suggest that homodimerization contributes more to self-renewal than Sox2 heterodimerization, the residual detectable heterodimerization prevents definitive conclusions being drawn.

To determine whether mutagenesis affected Nanog protein structure, comparative protease digestion of Nanog and NanogW10A was performed. A similar digestion profile for both proteins is suggestive of an unaltered overall structure upon tryptophan mutagenesis. Furthermore, these digests demonstrate that Nanog has a structural core extending from the start of the homeodomain to near to the C-terminus. The presence of an unstructured N-terminus is consistent with results of protease digestion of a Nanog fragment comprising the N-terminal domain and the homeodomain [10]. Full-length Nanog gives a second resistant species starting at residue 76. This may be due to partial occlusion of the protease site at residue 101, immediately N-terminal to the homeodomain by folding of full-length Nanog. In this respect, it is notable that the 28- and 23-kDa protease-resistant fragments differ in size by 5 kDa. Since the N-termini of these fragments begin at residues 76 and 101, a difference in size of ~ 2.8 kDa, the size difference between the 28- and 23-kDa fragments may be due to a concomitant loss of the C-terminal 26 residues between 279 and 305. One possible explanation for this would be that unfolding of fragment 76–305 of Nanog simultaneously reveals protease cleavage sites at 101 and 279. This is the first description of a structural entity within Nanog that extends beyond the homeodomain. The presence of this element is intriguing given that the sequence of the WR is rich in prolines and residues with hydroxyl or amide groups, residues that are enriched in intrinsically disordered regions of proteins [22]. It is therefore likely that tryptophans are the dominant structural element within the WR. Our finding that Nanog homodimerization requires interactions between tryptophans on each dimer subunit favors the view that the interaction occurs by aromatic stacking of tryptophan side chains. The complete lack of charged residues within the WR may favor hydrophobic interactions at the dimerization interface. Moreover, the reversible introduction of charged residues within the WR could be a mechanism to regulate the strength of hydrophobic surface interactions. This could be achieved by post-translational modification of hydroxyl groups by, for example, phosphorylation. Binding interfaces of homo and hetero-oligomeric complexes are enriched for serine and threonine [23], and there is a strong tendency for sites of phosphorylation to be located on binding surfaces [24]. Furthermore, the level of conservation of phosphorylation sites at binding surfaces exceeds that of phosphorylation sites that are not part of binding interfaces [24]. Thus, the fact that serines and threonines of the WR are highly conserved is noteworthy. This may indicate the existence of a system for regulating Nanog dimerization by modulating the strength of aromatic–aromatic interactions through phosphorylation of adjacent residues within the WR. Further investigation of the biochemistry of this intriguing protein sequence may provide additional insights into the molecular details of regulation of the transcriptional network at the core of ES cell self-renewal.

## Methods

### DNA constructs

Tryptophan mutants were constructed by replacement of wild-type sequence with DNA encoding the WR with the requisite mutations (Genscript, USA). The NanogW10A mutation was made by insertion of a synthetic sequence between two SexAI sites in the coding sequence of Nanog in a pET15 background. The mutated sequence was subcloned into pPyCAGIP as a BstXI fragment. This synthetic sequence also contained a silent mutation which introduced a NheI site just upstream of the start of the tryptophan repeat. All subsequent WR mutants were constructed by inserting synthetic DNAs directly into pPyCAGIP between the NheI site and the NotI site flanking the 3′ end of the Nanog coding sequence.

### Expression and purification of recombinant proteins

rNanog and rNanogW10A were expressed, purified and refolded as previously described [13].

### Cell transfections and self-renewal assays

For assessment of function of Nanog mutants, ES cells were transfected and processed as described [25]. Twelve days after transfection cells were stained with a leukocyte alkaline phosphatase kit (Sigma). For co-immunoprecipitations, cells were transfected as described [13].

### Immunoprecipitations

Immunoprecipitations were performed on nuclear extract prepared by removal of cytoplasm by swelling in 20mM Pipes (pH8.0), 85mM KCl and 0.5% NP-40 and salt extraction of nuclei in 20mM Hepes (pH7.6), 350mM KCl, 0.2mM EDTA, 1.5mM MgCl_2_, 20% glycerol and 0.2% NP-40. Proteins were precipitated using 5μg of antibody and protein–antibody complexes purified with Protein G. Complexes were eluted from beads by boiling in sample buffer [26].

### Analytical ultracentrifugation

Sedimentation velocity analysis and sedimentation equilibrium analysis were performed in a Beckman XL-A analytical ultracentrifuge: analyzing consecutively samples loaded either at original concentration (5.2 mg/ml) or after dilution 1:39. For sedimentation velocity analysis at 17,000 rpm during the initial period (1 h) of the run, where a solution plateau remained in the central region of the solution column, scans at 279 nm were taken at 4-min intervals and analyzed using the software SEDFIT [27] with a resolution setting of 200 and an *F* value of 0.2. This latter value meant that no regularization was employed. This gives high sensitivity to the presence of multiple components and enables sharply defined peaks to be seen, although diffusion will be extensive at the relatively low rotor speed employed. A default value of 1.20 for the frictional ratio enabled stable fits to be found, although the size of the data set was limited.

### Limited proteolysis

Limited proteolysis was performed with restriction grade trypsin or thermolysin (Roche). Digests were performed with a ratio of Nanog/protease of 600:1 (w/w) at 37 °C for 40 min. Reactions were terminated by addition of an equal volume of 2 × Laemli buffer and boiling for 5 min. Reactions performed in the presence of DNA were performed with DNA in a 5-fold molar excess over the protein. The DNA oligos used were 20mers based on the oligonucleotide sequence shown to bind to Nanog by SELEX [6]. N-terminal sequencing was performed at the LIGHT Laboratories Faculty of Biological Sciences at Leeds University using standard Edman degradation.
